# Supplementary material for: Inner core composition paradox revealed by sound velocities of Fe and Fe-Si alloy
Source: Nat Commun. 2022 Feb 1;13:616. doi: 10.1038/s41467-022-28255-2 (PMC8807611; doi:10.1038/s41467-022-28255-2)
Supplement: Supplementary file 1 — Supplementary Information [file 41467_2022_28255_MOESM1_ESM.pdf]

SUPPLEMENTARY INFORMATION

Inner core composition paradox revealed by sound velocities of Fe and Fe-Si alloy

Haijun Huang[1], Lili Fan[1], Xun Liu[1], Feng Xu[1], Ye Wu[1], Gang Yang[1], Chunwei Leng[1], Qingsong Wang[2], Jidong Weng[2], Xiang Wang[2], Lingcang Cai[2], Yingwei Fei[3*]

[1] School of Sciences, Wuhan University of Technology, Wuhan, Hubei 430070, China

[2] National Key Laboratory of Shock Wave and Detonation Physics, Institute of Fluid Physics, China Academy of Engineering Physics, Mianyang, Sichuan 621900, China

[3]Earth and Planets Laboratory, Carnegie Institution for Science, Washington, DC 20015, USA
*Corresponding author: Yingwei Fei (yfei@carnegiescience.edu)

This PDF file includes:

Supplementary Figures 1-12
Supplementary Tables 1-4
Supplementary References

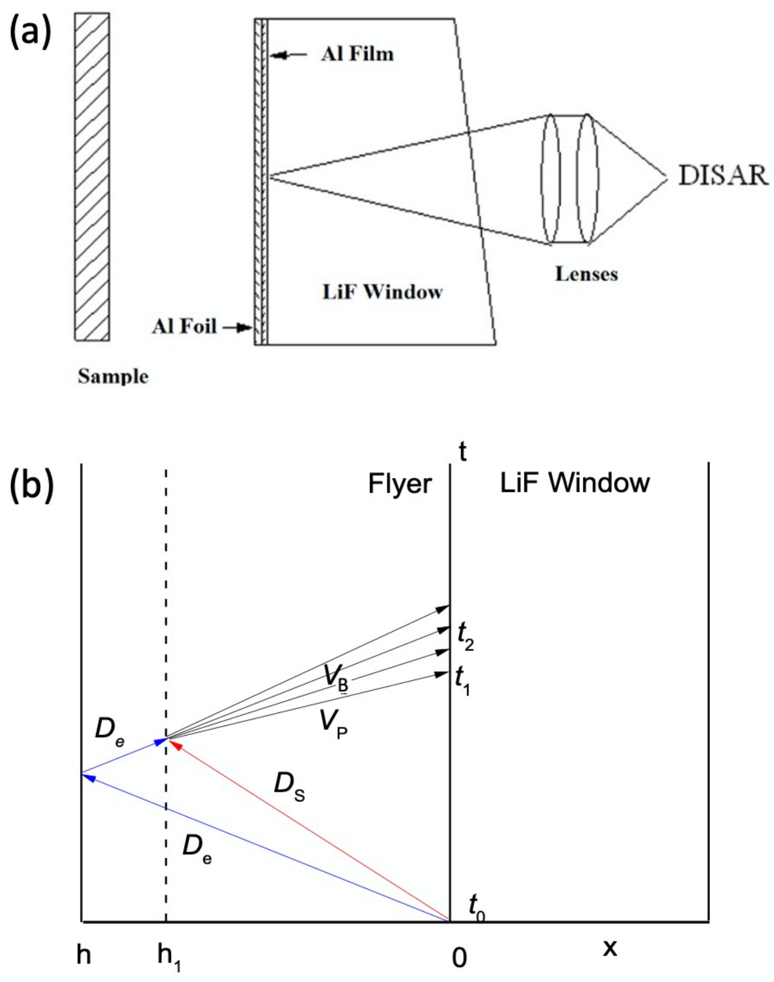

Supplementary Fig. 1. **Experimental setup and Lagrangian distance-time diagram for the reverse-impact experiments. (a)** Schematic of the experimental configuration for the reverse-impact experiments. The sample is used as a flyer to impact the LiF window. In front of the LiF window, aluminum (Al) film was coated with thickness of 3 μm. An 8-μm aluminum foil was mounted on the Al film with epoxy. The particle velocity at the interface between Al film and LiF interface was measured by DISAR. **(b)** Lagrangian distance-time diagram for the reverse-impact experiments. h is the thickness of the flyer. D_e is the elastic precursor velocity, D_s is the shockwave velocity, and V_P is the compressional wave velocity under compressed state. t_0 and t_1 are the arrival times of the shock wave and rarefaction wave at the interface, respectively. t_2 represents the time at the elastic-plastic transition point which allows determination of the bulk sound velocity V_B during unloading. h_1 is the position where the reflected precursor and direct shock interact.

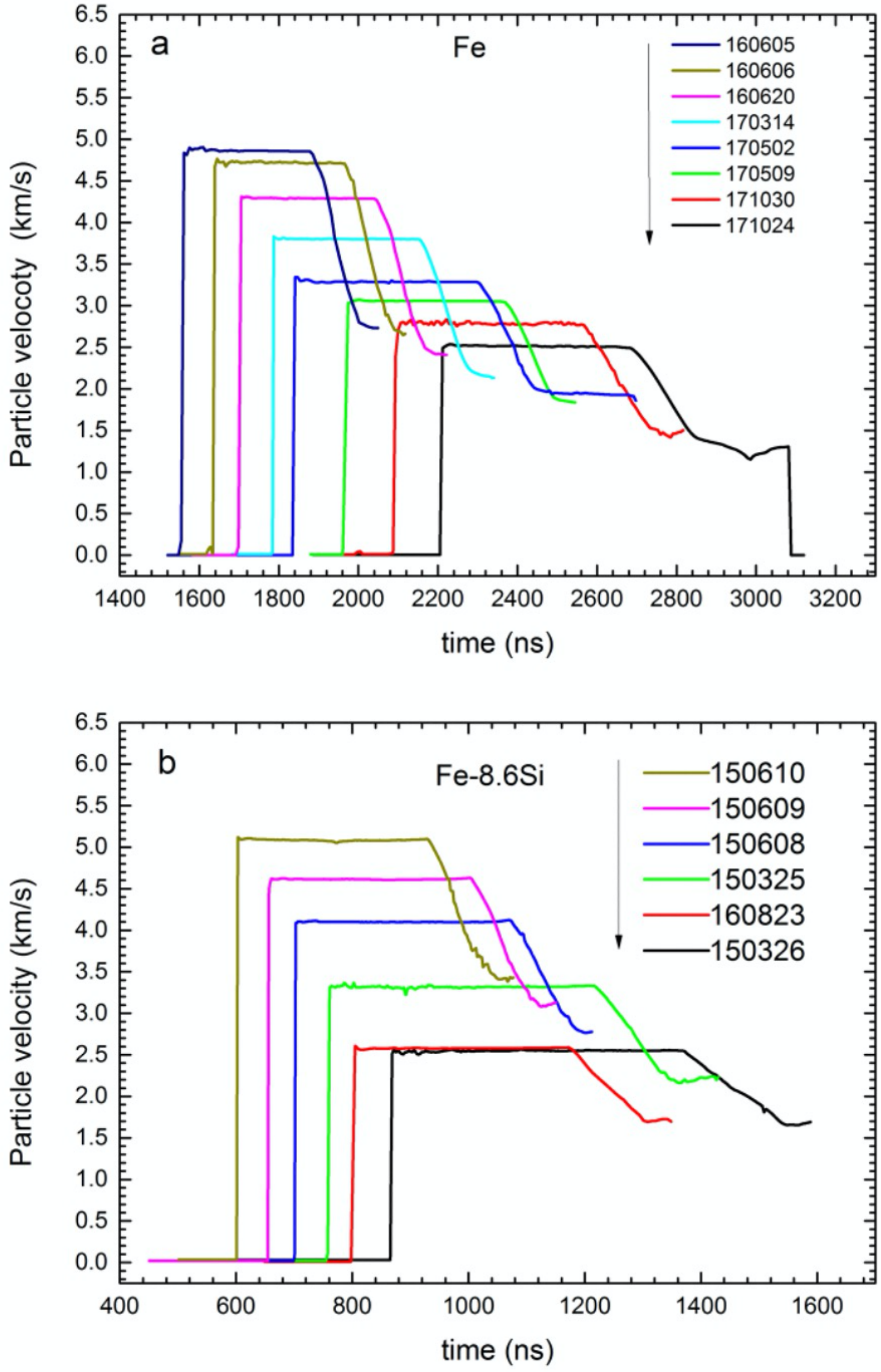

Supplementary Fig. 2. **Recorded particle velocity histories from the reverse-impact experiments. (a)** The signal for each shot was recorded by DISAR and a total of eight shots were performed for Fe at shock pressures from 57 GPa to 151 GPa. **(b)** The signal for each shot was recorded by DISAR and a total of six shots were performed for Fe-8.6Si alloy at shock pressures from 58 GPa to 162 GPa.

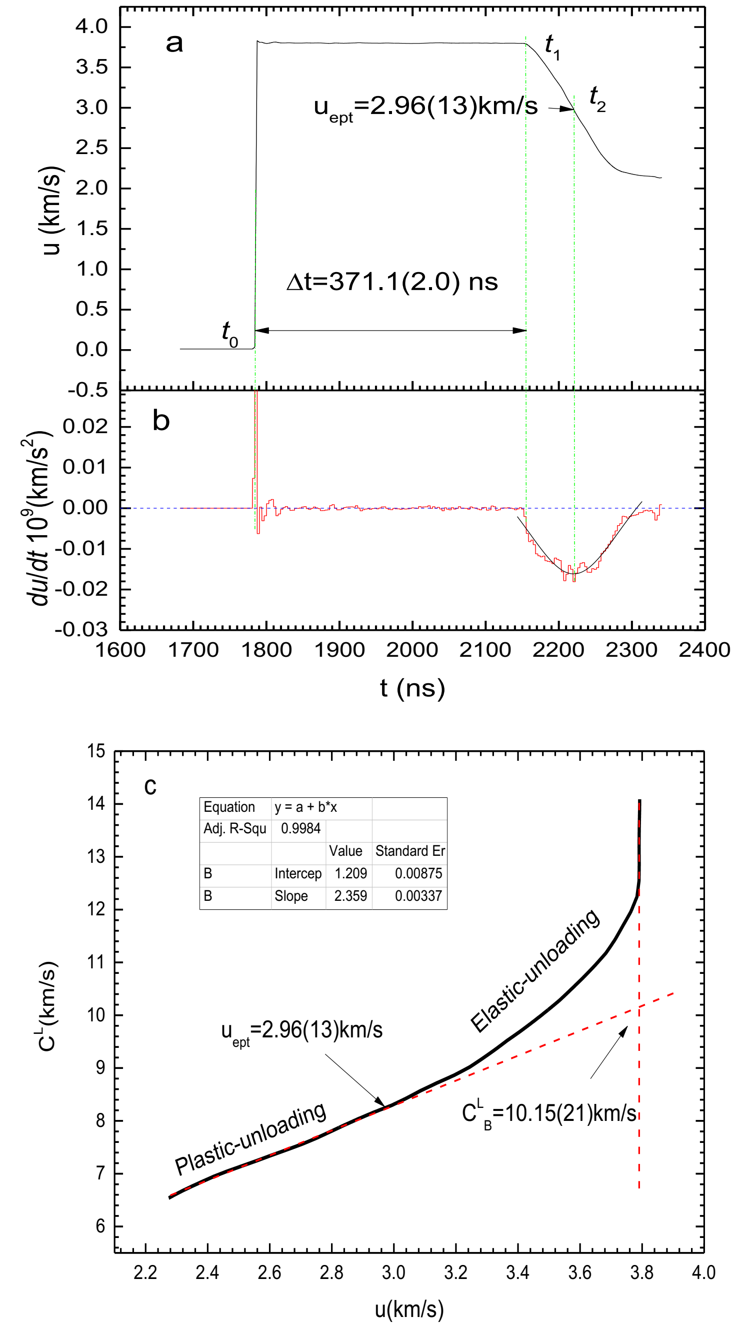

Supplementary Fig. 3. **Procedure for determination of the sound velocities from particle velocity history. (a**) Particle velocity history *u(t)* for experiment 170314. t_0 and t_1 correspond to the arrival times of the shock wave front and rarefaction wave at the interface between the Al film and LiF window, respectively. t_2 corresponds to the elastic-plastic transition (EPT) point during unloading. **(b)** The derivative of particle velocity with respect to time, du/dt. At t_1, du/dt decreases rapidly and then reaches a minimum value due to the EPT at t_2. **(c)** The Lagrange sound velocity vs the particle velocity. The intersection of the dashed lines represents the Lagrangian bulk sound just before unloading.

(a)

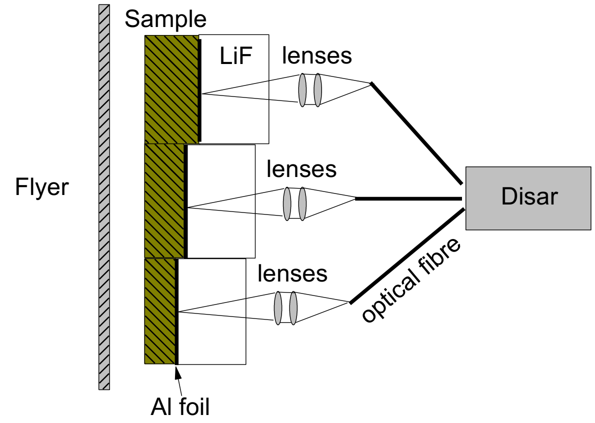

(b)

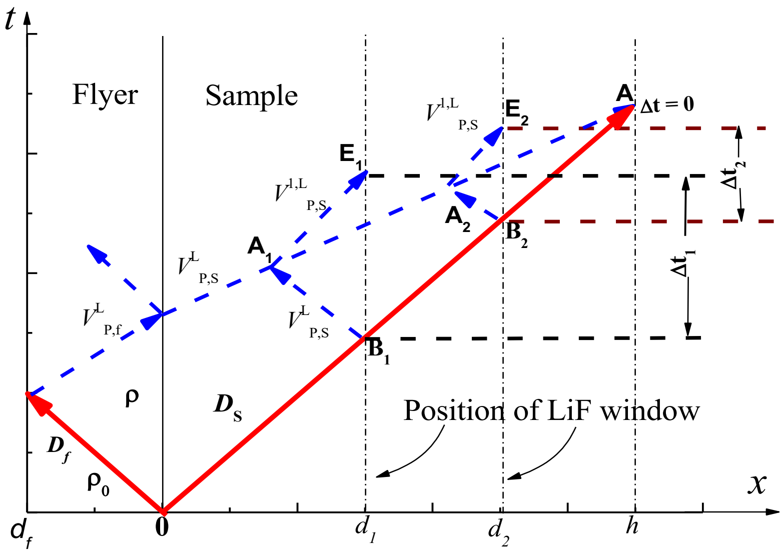

Supplementary Fig. 4. **Experimental setup and Lagrangian distance-time diagram for the optical analyzer experiments. (a)** Schematic of experimental setup for the optical analyzer technique (OAT) using multi-sample method. Samples with three different thicknesses were used to obtain the catch-up thickness of the sample. DISAR was used to measure the particle velocity at the aluminum film/LiF interface. **(b)** The Lagrangian distance-time diagram for the OAT. d_f is the thickness of the flyer. d_1 and d_2 represent the different thicknesses of the samples. The solid lines represent the shock waves D, and the dashed lines represent the rarefaction wave with velocity V_P. The superscript L indicates the Lagrangian coordinates. The subscripts f and s represent the flyer and sample, respectively. The vertical dash lines indicate the positions of the LiF window.

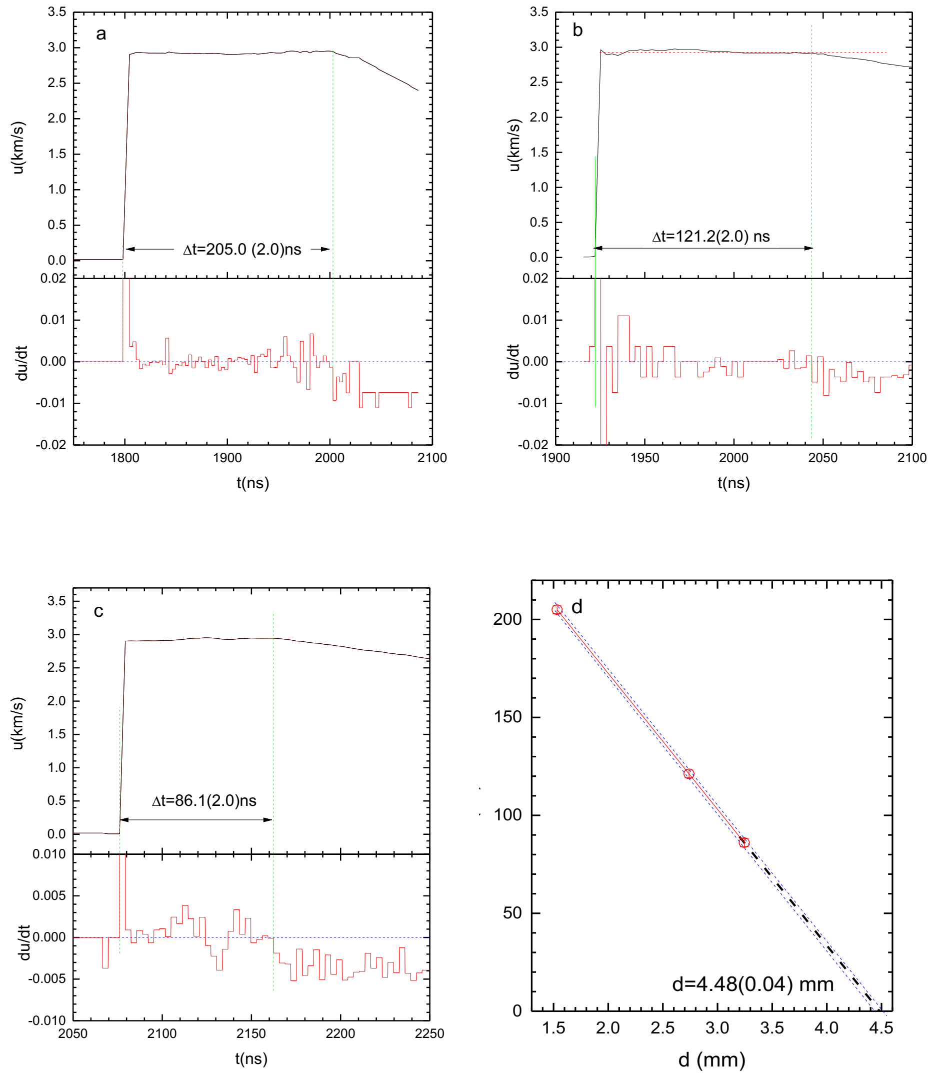

Supplementary Fig. 5. **Procedure for determination of the catch-up thickness from three samples with different thicknesses. (a-c**) The particle velocity histories (experiment 180102) in the three samples with different thicknesses at 126 GPa. The time intervals were measured by DISAR for the samples with thicknesses of 1.532 (±0.002) mm, 2.739 (±0.002) mm and 3.244 (±0.002) mm. (**d**) The time interval vs thickness of the sample. A linear fit to the data yielded a catch-up thickness of 4.48 (±0.04) mm.

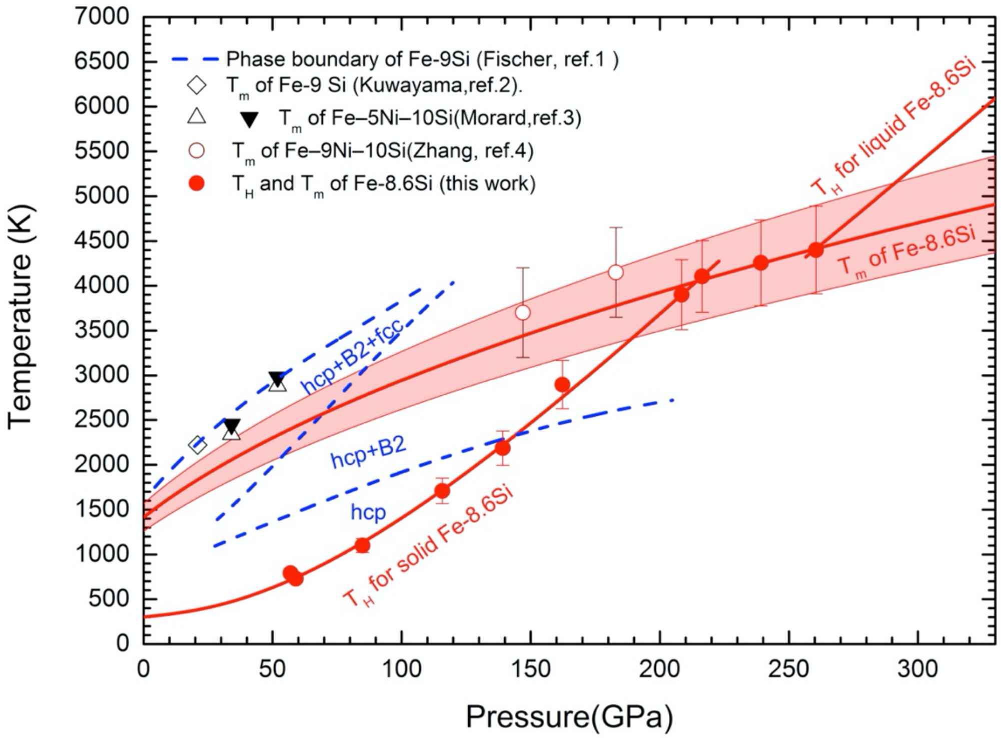

Supplementary Fig. 6. **Comparison of melting temperatures for Fe-Si alloys at high pressure.** Dash lines represent the phase boundary of Fe-9Si by static compression [1]. The open diamond represents the melting temperature of Fe-9Si at 21 GPa [2]. Up and down triangles represent the up and low bounds of melting temperature of Fe-5Ni-10Si [3]. Open circles represent the melting temperature of Fe-9Ni-10Si [4]. The shock temperatures for this study were calculated from thermodynamic model.

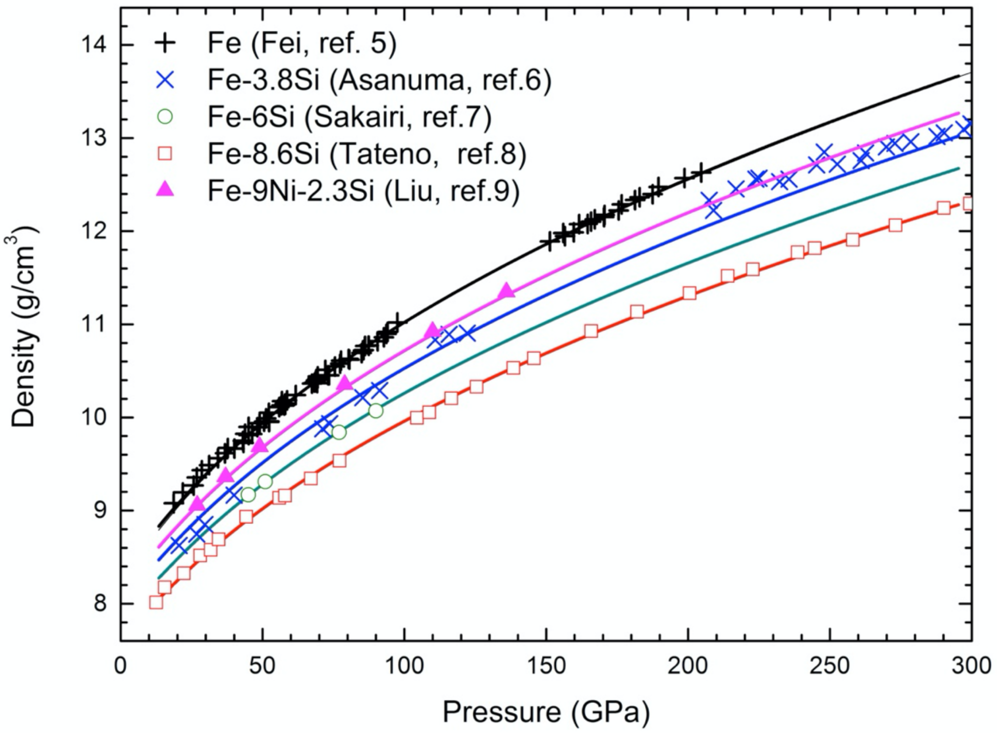

Supplementary Fig. 7. **The compositional effect on the equations of state of Fe-Si alloys at room temperature**. The symbols represent the measurements by static compression, and the solid lines represent the calculated results based on ideal mixing model with the data of the endmembers Fe and Fe-8.6Si.

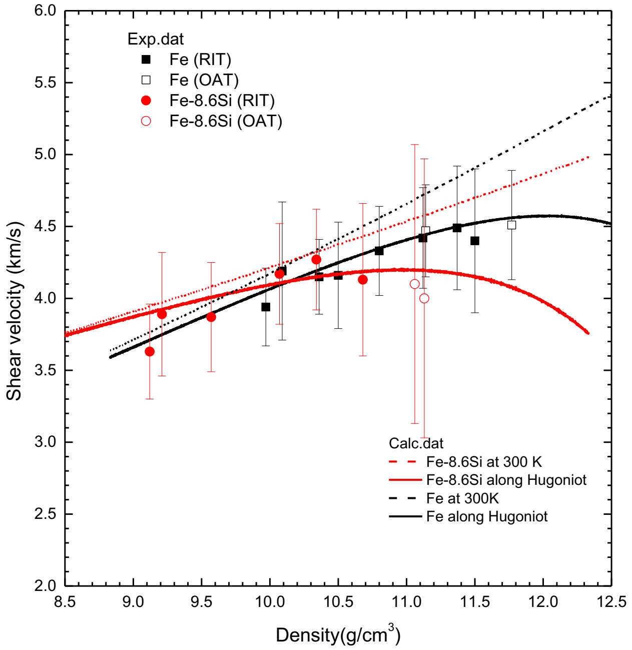

Supplementary Fig. 8. **Shear velocity of Fe and Fe-8.6Si as a function of density along Hugoniot and 300 K isotherm.** Solid and open squares represent the measured Hugoniot data for Fe from the RIT and the OAT, respectively. Solid and open circles are the Hugoniot data for Fe-8.6Si alloy from the RIT and the OAT, respectively. The solid and dashed lines represent the calculated results along the Hugoniot and 300 K isotherm, respectively.

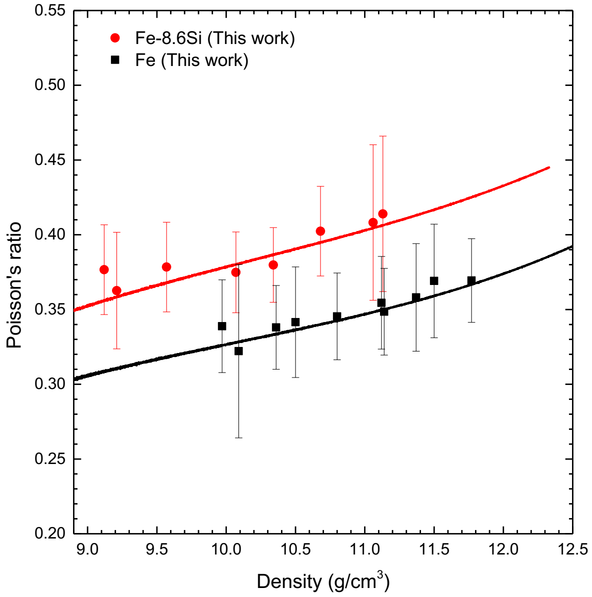

Supplementary Fig. 9. **The Poisson's ratio σ of Fe and Fe-8.6Si as a function of density under the shock compression.** The symbols represent experimental measurements, whereas the lines are calculated results. At the same density, σ of Fe-8.6Si is ~16% larger than that of pure Fe.

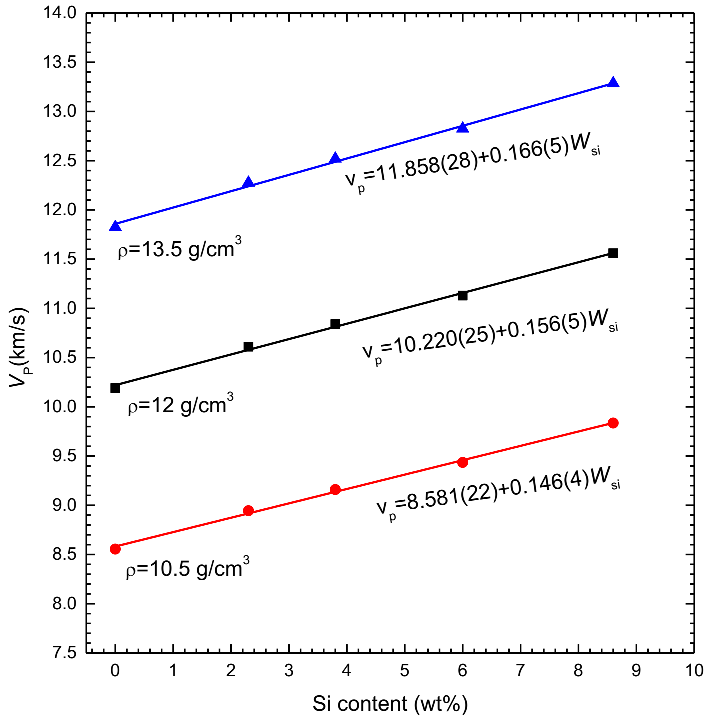

Supplementary Fig. 10. **The effect of Si content (W_{Si}) on the compressional velocity of Fe-Si alloy.** At a constant density, V_p of the Fe-Si alloy increases linearly with $W_{Si,}$ and the average slope is nearly constant, dV_p /dW_{Si} =0.156(10) km/s. The intercepts of solid lines also increase with density ρ.

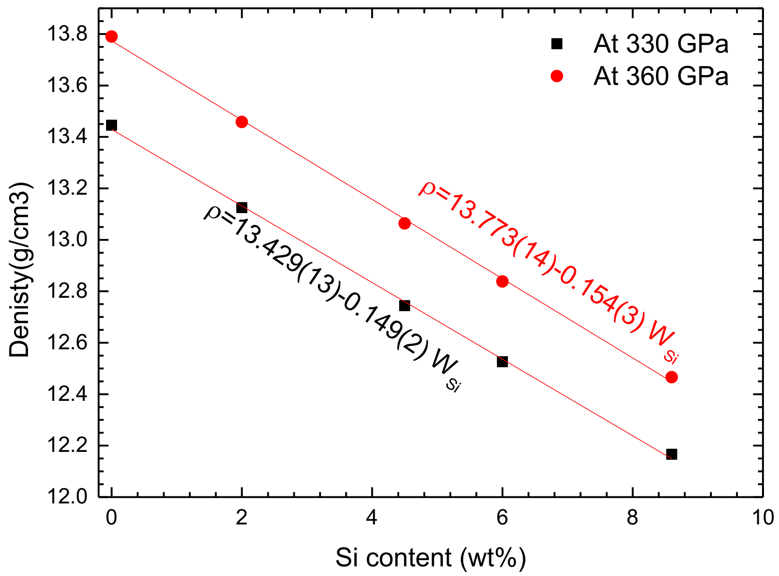

Supplementary Fig. 11. **The effect of Si content (W_{Si}) on the density of Fe-Si alloy.** At a constant pressure, the density of Fe-Si alloy decreases linearly with $W_{Si,}$. Data show density changes with Si content at 330 and 360 GPa.

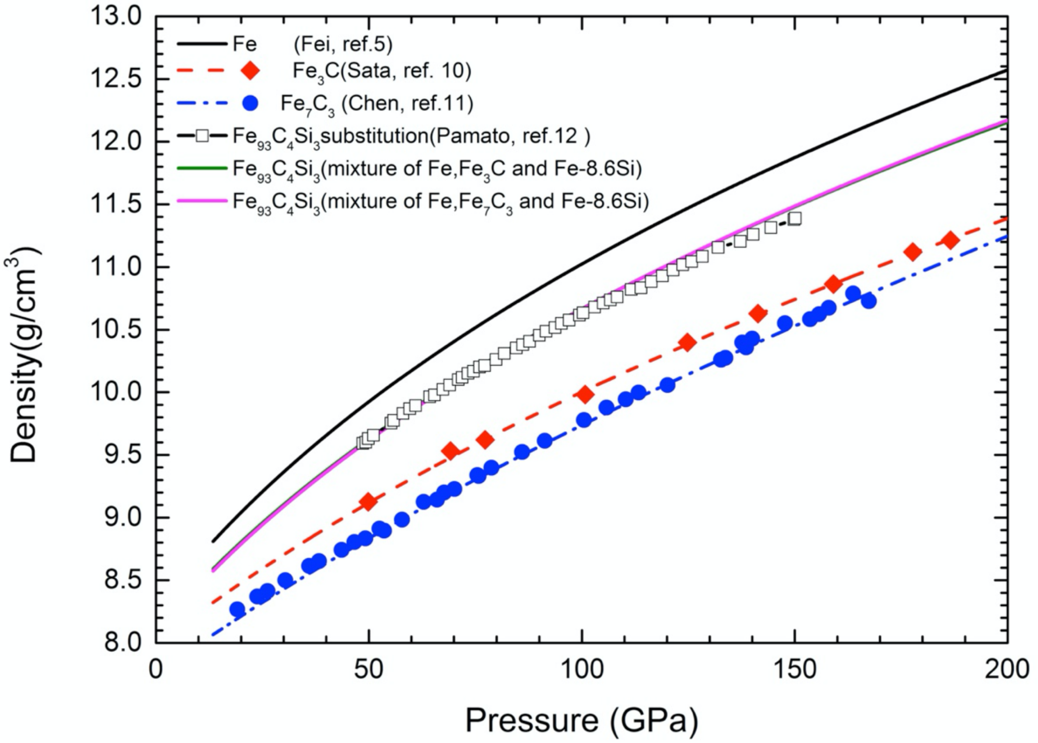

Supplementary Fig. 12. **Compositional effect on the equation of state in the Fe-Si-C system.** The symbols represent the measurements by static compression, and the solid lines represent the calculated results based on an ideal mixing model using data from the endmembers Fe, Fe-8.6Si and Fe_7C_3.

Supplementary Table 1. Sound velocity measurements of Fe and Fe-8.6Si using the RIT. ρ_0 is the initial density. *h is* the sample thickness. *W is* the impact velocity. *u is the* particle velocity. D_S is the shock velocity. *P* is pressure. ρ is the compressed density. V_P, V_B, V_S and σ are compressional, bulk, shear velocity and Poisson's ratio. *T* is the calculated shock temperature.

Shot number	Sample	ρ_0 (g/cm^3)	*h* (cm)	*W* (km/s)	*u* (km/s)	D_S (km/s)	*P* (GPa)	ρ (g/cm^3)	V_P (km/s)	V_B (km/s)	V_S (km/s)	σ	*T* (K)
171024	Fe	7.859 ± 0.004	1.806 ± 0.002	3.749 ± 0.005	1.237 ± 0.007	5.845 ±0.054	56.8 ±0.6	9.97 ±0.03	7.98 ±0.14	6.55 ±0.14	3.94 ±0.27	0.338 ±0.031	1073 ± 56
171030	Fe	7.855 ± 0.004	1.838 ± 0.002	4.138 ± 0.004	1.360 ± 0.014	6.130 ±0.102	65.5 ±1.3	10.09 ±0.06	8.18 ±0.25	6.60 ±0.25	4.19 ±0.48	0.322 ±0.058	1167 ± 89
170509	Fe	7.864 ± 0.003	1.622 ± 0.003	4.560 ± 0.005	1.513 ± 0.005	6.284 ±0.027	74.8 ±0.4	10.36 ±0.02	8.39 ±0.13	6.88 ±0.13	4.15 ±0.26	0.338 ±0.028	1416 ± 80
170502	Fe	7.868 ± 0.004	1.922 ± 0.002	4.920 ± 0.005	1.632 ± 0.010	6.505 ±0.066	83.5 ±1.0	10.50 ±0.04	8.48 ±0.18	7.00 ±0.18	4.16 ±0.37	0.341 ±0.037	1570 ± 110
170314	Fe	7.848 ± 0.004	1.645 ± 0.004	5.690 ± 0.005	1.896 ± 0.006	6.939 ±0.029	103.3 ±0.5	10.80 ±0.02	8.91 ±0.15	7.37 ±0.15	4.33 ±0.31	0.345 ±0.029	1960 ± 120
160620	Fe	7.867 ± 0.004	1.634 ± 0.002	6.440 ± 0.005	2.150 ± 0.006	7.354 ±0.033	124.4 ±0.7	11.12 ±0.03	9.31 ±0.17	7.78 ±0.17	4.42 ±0.35	0.354 ±0.031	2477 ± 170
160606	Fe	7.853 ± 0.003	1.641 ± 0.003	7.090 ± 0.005	2.379 ± 0.010	7.692 ±0.056	143.7 ±1.2	11.37 ±0.04	9.55 ±0.21	8.02 ±0.21	4.49 ±0.43	0.358 ±0.036	2966 ± 225
160605	Fe	7.861 ± 0.005	1.645 ± 0.002	7.320 ± 0.005	2.463 ± 0.012	7.783 ±0.066	150.7 ±1.5	11.50 ±0.05	9.66 ±0.23	8.22 ±0.23	4.40 ±0.50	0.369 ±0.038	3250 ± 268
150326	Fe-8.6Si	7.385 ± 0.004	1.985 ± 0.002	3.792 ± 0.005	1.246 ± 0.008	6.292 ±0.081	57.9 ±0.9	9.21 ±0.03	8.38 ±0.20	7.07 ±0.20	3.89 ±0.43	0.363 ±0.039	790 ± 44
160823	Fe-8.6Si	7.362 ± 0.004	1.463 ± 0.002	3.830 ± 0.005	1.245 ± 0.011	6.453 ±0.091	59.1 ±0.6	9.12 ±0.02	8.16 ±0.15	7.00 ±0.15	3.63 ±0.33	0.377 ±0.030	730 ± 40
150325	Fe-8.6Si	7.357 ± 0.004	1.992 ± 0.003	4.960 ± 0.007	1.634 ± 0.008	7.066 ±0.064	84.9 ±0.9	9.57 ±0.03	8.75 ±0.17	7.52 ±0.17	3.87 ±0.38	0.378 ±0.030	1099 ± 78
150608	Fe-8.6Si	7.385 ± 0.004	1.769 ± 0.003	6.139 ± 0.006	2.044 ± 0.007	7.676 ±0.041	115.8 ±0.7	10.07 ±0.04	9.32 ±0.16	7.97 ±0.16	4.17 ±0.35	0.374 ±0.027	1709 ± 142
150609	Fe-8.6Si	7.378 ± 0.004	1.769 ± 0.002	6.940 ± 0.007	2.325 ± 0.011	8.114 ±0.035	139.2 ±0.7	10.34 ±0.02	9.70 ±0.17	8.35 ±0.17	4.27 ±0.35	0.379 ±0.025	2186 ± 192
150610	Fe-8.6Si	7.391 ± 0.004	1.775 ± 0.003	7.692 ± 0.006	2.600 ± 0.011	8.445 ±0.058	162.3 ±1.3	10.68 ±0.04	10.22 ±0.21	9.04 ±0.21	4.13 ±0.53	0.403 ±0.030	2896 ± 270

Supplementary Table 2. Sound velocity measurements of Fe and Fe-8.6Si using OAT. *W* is the impact velocity. *P* is pressure. ρ is the compressed density. $V_{P,}$, V_B, V_S and σ are compressional, bulk and shear velocity, and Poisson's ratio. *T* is the calculated shock temperature. V_P is directly measured. V_B *is* calculated based on equation of state. V_s and σ are obtained via calculated V_B and measured V_P.

Shot number	Impactor	Sample	*W* (km/s)	*P* (GPa)	ρ (g/cm^3)	*R*	V_P (km/s)	V_B (km/s)	V_S (km/s)	σ	*T* (K)
180102	Fe	Fe	4.363 ± 0.004	126.3 ± 0.7	11.14 ± 0.03	3.55 ± 0.03	9.27 ± 0.08	7.70 ± 0.23	4.47 ± 0.32	0.348 ± 0.029	2521 ± 180
171025	Fe	Fe	5.530 ± 0.004	180.1 ± 0.9	11.77 ± 0.03	3.53 ± 0.03	9.91 ± 0.08	8.43 ± 0.26	4.51 ± 0.38	0.369 ± 0.028	3919 ± 273
170623	Fe	Fe	6.120 ± 0.005	210.5 ± 1.1	12.06 ± 0.03	3.77 ± 0.05	9.81 ± 0.10	8.79 ± 0.27	3.78 ± 0.51	0.413 ± 0.028	4758 ± 330
170523	Fe	Fe	6.550 ± 0.005	234.0 ±1.2	12.26 ± 0.03	3.96 ± 0.05	9.76 ± 0.10	9.06 ± 0.27	3.14 ± 0.63	0.442 ± 0.026	5417 ± 372
150625	Ta	Fe-8.6Si	5.144 ± 0.004	208.5 ±3.7	11.06 ± 0.09	6.00 ± 0.03	10.41 ± 0.38	9.27 ± 0.38	4.10 ± 0.97	0.408 ± 0.052	3900 ± 390
160116	Ta	Fe-8.6Si	5.274 ± 0.005	216.4 ±3.8	11.13 ± 0.09	6.03 ± 0.04	10.44 ± 0.39	9.36 ± 0.39	4.00 ± 0.97	0.414 ± 0.052	4105 ± 400
160304	Ta	Fe-8.6Si	5.640 ± 0.004	239.2 ±4.2	11.32 ± 0.10	6.30 ± 0.03	10.31 ± 0. 44	9.62 ± 0.50	3.21 ± 0.97	0.446 ±0. 054	4255 ± 480
151218	Ta	Fe-8.6Si	5.967 ± 0.004	260.5 ±4.6	11.48 ± 0.11	7.23 ± 0.04	9..67 ± 0. 34	9.67 ± 0.34	0	0.5	4400 ± 490

Supplementary Table 3. The thermodynamic parameters for Fe , Fe-8.6Si and Fe_7C_3. ρ_{0a} and ρ_{0h} represent the density at ambient condition and high pressure condition, respectively.

Alloy	ρ_{0a} g/cm^3	C_0 km/s	λ	ρ_{0h} g/cm^3	K_0 GPa	K'_0	γ_0	q	β_0 J/(Kg.K^2)	κ	γ_e
Fe-8.6Si	7.386[a] ± 0.021	4.603 [a] ± 0.101	1.505 [a] ± 0.037	7.578 [a] ± 0.050	190.8 [a] ± 6.7	4.74 [a] ± 0.28	1.65 [a] ± 0.1	1 [a] ± 0.1	0.091 [b] ± 0.01	1.34 [b] ± 0.01	1.83 [a]
Fe	7.850[c] ± 0.002	3.935 [c] ± 0.029	1.578 [c] ± 0.010	8.269[d] --	181.6 ±3.2	4.58 ± 0.07	1.74[d] ± 0.1	0.78 [d] ± 0.1	0.091 [c] ± 0.01	1.34[d] ± 0.01	2[e]
Fe_7C_3	---	---	---	7.75 [f] ± 0.02	307 [f] ± 6	3.2 [f] ± 0.1	2.57 [g] ± 0.05	2.2 [g] ± 0.5	--	--	--

[a] Huang et al. (ref. 13); [b] Boness et al. (ref. 14); [c] Brown et al. (ref. 15); [d] Fei et al. (ref. 5);[e] Anderson (ref. 16).

[f] Chen et al. (ref. 11), [g] Nakajima et al. (ref. 17)

Supplementary Table 4. The calculated densities and sound velocities for Fe, Fe-8.6Si, Fe-4.5Si, Fe_7C_3 and Fe-1Si-5C assuming a temperature of 5440 K at the inner core boundary.

P GPa	Fe			*Fe-8.6Si*			*Fe-4.5Si*			Fe_7C_3			*Fe-1Si-5C*		
	ρ g/cm3	V_P km/s	V_S *km/s*	ρ g/cm	V_P km/s	V_S *km/s*	ρ g/cm3	V_P km/s	V_S *km/s*	ρ g/cm3	V_P km/s	V_S *km/s*	ρ g/cm3	V_P km/s	V_S *km/s*
328.92	13.44	11.77	5.48	12.17	11.75	4.41	12.74	11.76	4.85	12.48	10.40	3.23	12.70	10.89	3.77
330.13	13.46	11.78	5.48	12.18	11.76	4.43	12.75	11.77	4.87	12.49	10.40	3.23	12.72	10.90	3.78
335.56	13.51	11.84	5.51	12.22	11.82	4.44	12.80	11.83	4.88	12.56	10.45	3.24	12.78	10.95	3.79
340.20	13.56	11.89	5.54	12.27	11.86	4.46	12.85	11.88	4.90	12.62	10.49	3.26	12.83	11.00	3.81
344.75	13.60	11.94	5.56	12.30	11.91	4.48	12.89	11.92	4.92	12.67	10.52	3.27	12.88	11.04	3.83
348.85	13.64	11.98	5.58	12.34	11.95	4.50	12.93	11.96	4.93	12.72	10.56	3.28	12.93	11.07	3.84
352.22	13.68	12.02	5.60	12.37	11.98	4.50	12.96	12.00	4.94	12.76	10.58	3.29	12.96	11.10	3.85
355.45	13.71	12.05	5.62	12.39	12.01	4.50	12.99	12.03	4.94	12.80	10.61	3.30	13.00	11.13	3.86
357.91	13.73	12.08	5.64	12.42	12.04	4.51	13.01	12.06	4.95	12.83	10.63	3.31	13.03	11.16	3.87
360.04	13.75	12.10	5.65	12.43	12.06	4.52	13.03	12.08	4.96	12.86	10.65	3.32	13.05	11.17	3.88
361.83	13.77	12.12	5.66	12.45	12.07	4.53	13.04	12.09	4.97	12.88	10.66	3.32	13.07	11.19	3.89
363.00	13.78	12.13	5.67	12.46	12.09	4.52	13.06	12.11	4.96	12.89	10.67	3.33	13.08	11.20	3.89
363.63	13.79	12.14	5.67	12.46	12.09	4.52	13.06	12.11	4.97	12.90	10.67	3.33	13.09	11.20	3.89
363.99	13.79	12.14	5.67	12.47	12.10	4.54	13.06	12.12	4.97	12.91	10.68	3.33	13.09	11.21	3.90
uncertainty	0.11	0.31	0.57	0.29	0.52	1.2	0.18	0.32	0.81	0.24	1.9	0.71	0.15	1.26	0.6

.

Supplementary References

1. Fischer, R. A. et al. Phase relations in the Fe–FeSi system at high pressures and temperatures. *Earth Planet. Sci. Lett.* **373**, 54-64 (2013).
2. Kuwayama, Y. & Hirose, K. Phase relations in the system Fe–FeSi at 21GPa. *Am. Mineral.* **89**, 273–276 (2004).
3. Morard, G., Andrault, D., Guignot, N., Siebert, J., Garbarino, G., & Antonangeli, D. Melting of Fe–Ni–Si and Fe–Ni–S alloys at megabar pressures: implications for the core–mantle boundary temperature. *Physics and Chemistry of Minerals* **38(10)**, 767-776 (2011).
4. Zhang, Y. et al. Experimental constraints on light elements in the earth's outer core. *Scientific Reports* **6**, 22473 (2016).
5. Fei, Y., Murphy, C., Shibazaki, Y., Shahar, A. & Huang, H. Thermal equation of state of hcp - iron: Constraint on the density deficit of earth's solid inner core. *Geophys. Res. Lett.* **43**, 6837-6843 (2016).
6. Asanuma, H. et al. Static compression of $Fe_{0.83}Ni_{0.09}Si_{0.08}$ alloy to 374 GPa and $Fe_{0.93}Si_{0.07}$ alloy to 252 GPa: Implications for the Earth's inner core. *Earth and Planetary Science Letters* **310(1-2)**, 113-118 (2011).
7. Sakairi, T. et al. Sound velocity measurements of hcp Fe-Si alloy at high pressure and high temperature by inelastic x-ray scattering. *Am. Mineral.* **103**, 85-90 (2018).
8. Tateno, S., Kuwayama, Y., Hirose, K. & Ohishi, Y. The structure of Fe–Si alloy in Earth's inner core. *Earth Planet. Sci. Lett.* **418**, 11–19(2015).
9. Liu, J. et al. Seismic parameters of hcp-Fe alloyed with Ni and Si in the Earth's inner core. *J. Geophys. Res.* **121**, 610-623 (2016).
10. Sata, N. et al. Compression of FeSi, Fe_3C, $Fe_{0.95}O$, and FeS under the core pressures and implication for light element in the Earth's core. *J. Geophys. Res.* **115**, B09204 (2010).
11. Chen, B. et al. Magneto-elastic coupling in compressed Fe_7C_3 supports carbon in Earth's inner core. *Geophys. Res. Lett.* **39**, L18301 (2012).

12. Pamato, M. G. et al. Equation of state of hcp Fe‐C‐Si alloys and the effect of C incorporation mechanism on the density of hcp Fe alloys at 300 K. *J. Geophys. Res*. **125**, e2020JB020159 (2020).
13. Huang, H. et al. Equation of state for shocked Fe‐8.6wt% Si up to 240 GPa and 4,670 K. *J. Geophys. Res*. **124**, 8300-8312 (2019).
14. Boness, D. A., Brown, J. M. & McMahan, A. K. The electronic thermodynamics of iron under Earth core conditions. *Phys. Earth Planet. Inter.* **42**(4), 227–240 (1986).
15. Brown, J. M., Fritz, J. N. & Hixson, R. S. Hugoniot data for iron. *Journal of Applied Physics* **88**, 5496-5498 (2000).
16. Anderson, O. L. The power balance at the core‐mantle boundary. *Phys. Earth Planet. Inter*. **131**, 1–17 (2002).
17. Nakajima, Y., Takahashi, E., Sata, N., Nishihara, Y., Hirose, K., Funakoshi, K. & Ohishi, Y. Thermoelastic property and high-pressure stability of Fe_7C_3: Implication for iron-carbide in the Earth's core. *Am. Mineral.* **96**, 1158–1165 (2011).
